# Decentralized Heart Failure Management in Neno, Malawi

**DOI:** 10.5334/gh.1210

**Published:** 2023-06-16

**Authors:** Bright G. D. Mailosi, Todd Ruderman, Sheila L. Klassen, Chiyembekezo Kachimanga, Moses Banda Aron, Medson Boti, Kenwood Kumwenda, Gene Bukhman, Adamson S. Muula, Ndaziona P. K. Banda, Gene F. Kwan

**Affiliations:** 1 Partners In Health/Abwenzi Pa Za Umoyo, Neno, Malawi; 2 Partners In Health, Boston, MA, 02199, USA; 3 Center for Integration Science, Division of Global Health Equity and Division of Cardiovascular Medicine, Brigham and Women’s Hospital, USA; 4 Program in Global NCDs and Social Change, Department of Global Health and Social Medicine, Harvard Medical School, Boston, MA, USA; 5 Department of Community and Environmental Health, School of Global and Public Health, Kamuzu University of Health Sciences, Blantyre, Malawi; 6 Kamuzu College of Health Sciences, Malawi; 7 Section of Cardiovascular Medicine, Boston University School of Medicine, Boston, MA USA 02118, USA

**Keywords:** Heart Failure, Focused cardiac ultrasound, Task-shifting, Malawi

## Abstract

**Background:**

Cardiovascular disease (CVD) is a major cause of death in Malawi. In rural districts, heart failure (HF) care is limited and provided by non-physicians. The causes and patient outcomes of HF in rural Africa are largely unknown. In our study, non-physician providers performed focused cardiac ultrasound (FOCUS) for HF diagnosis and longitudinal clinical follow-up in Neno, Malawi.

**Objectives:**

We described the clinical characteristics, HF categories, and outcomes of patients presenting with HF in chronic care clinics in Neno, Malawi.

**Methods:**

Between November 2018 and March 2021, non-physician providers performed FOCUS for diagnosis and longitudinal follow-up in an outpatient chronic disease clinic in rural Malawi. A retrospective chart review was performed for HF diagnostic categories, change in clinical status between enrollment and follow-up, and clinical outcomes. For study purposes, cardiologists reviewed all available ultrasound images.

**Results:**

There were 178 patients with HF, a median age of 67 years (IQR 44 – 75), and 103 (58%) women. During the study period, patients were enrolled for a mean of 11.5 months (IQR 5.1–16.5), after which 139 (78%) were alive and in care. The most common diagnostic categories by cardiac ultrasound were hypertensive heart disease (36%), cardiomyopathy (26%), and rheumatic, valvular or congenital heart disease (12.3%).

At follow-up, the proportion of New York Heart Association (NYHA) class I patients increased from 24% to 50% (p < 0.001; 95% CI: 31.5 – 16.4), and symptoms of orthopnea, edema, fatigue, hypervolemia, and bibasilar crackles all decreased (p < 0.05).

**Conclusion:**

Hypertensive heart disease and cardiomyopathy are the predominant causes of HF in this elderly cohort in rural Malawi. Trained non-physician providers can successfully manage HF to improve symptoms and clinical outcomes in limited resource areas. Similar care models could improve healthcare access in other rural African settings.

## Introduction

Non-communicable diseases (NCDs) account for 32% of all deaths in Malawi [1,2], 10% of which are due to cardiovascular disease [1]. In Malawi, heart failure (HF) has been previously described in urban settings [3], while rural data is not available despite 84% of the population residing in rural areas [4].

Malawi is a landlocked Sub-Saharan African country bordering Tanzania, Zambia, and Mozambique [5]. Ranked as one of the poorest countries in the world, Malawi has a population of 19.5 million people, which is expected to double by 2038 [5]. The country has an agrarian economy, with a GDP per capita of $522.96 in 2022 [6]. Over two-thirds of the population (73.2%) live below $1.90 a day [5].

Access to NCD care in Malawi is limited – especially in primary care, which serves as the first point of contact for people living in rural areas. This is evidenced by the lack of first-line antihypertensive medications in health centers and minimal inconsistent supply at district-level hospitals [7]. Poor population awareness of NCDs, long distances to health facilities, and prevalent use of herbal and traditional medicine all contribute to low population use of NCD services [8]. At the time of this study, Malawi only has one pediatric cardiologist, illustrating the challenges in the provision of specialized cardiovascular disease care for a population of 19 million.

**Figure 1:**
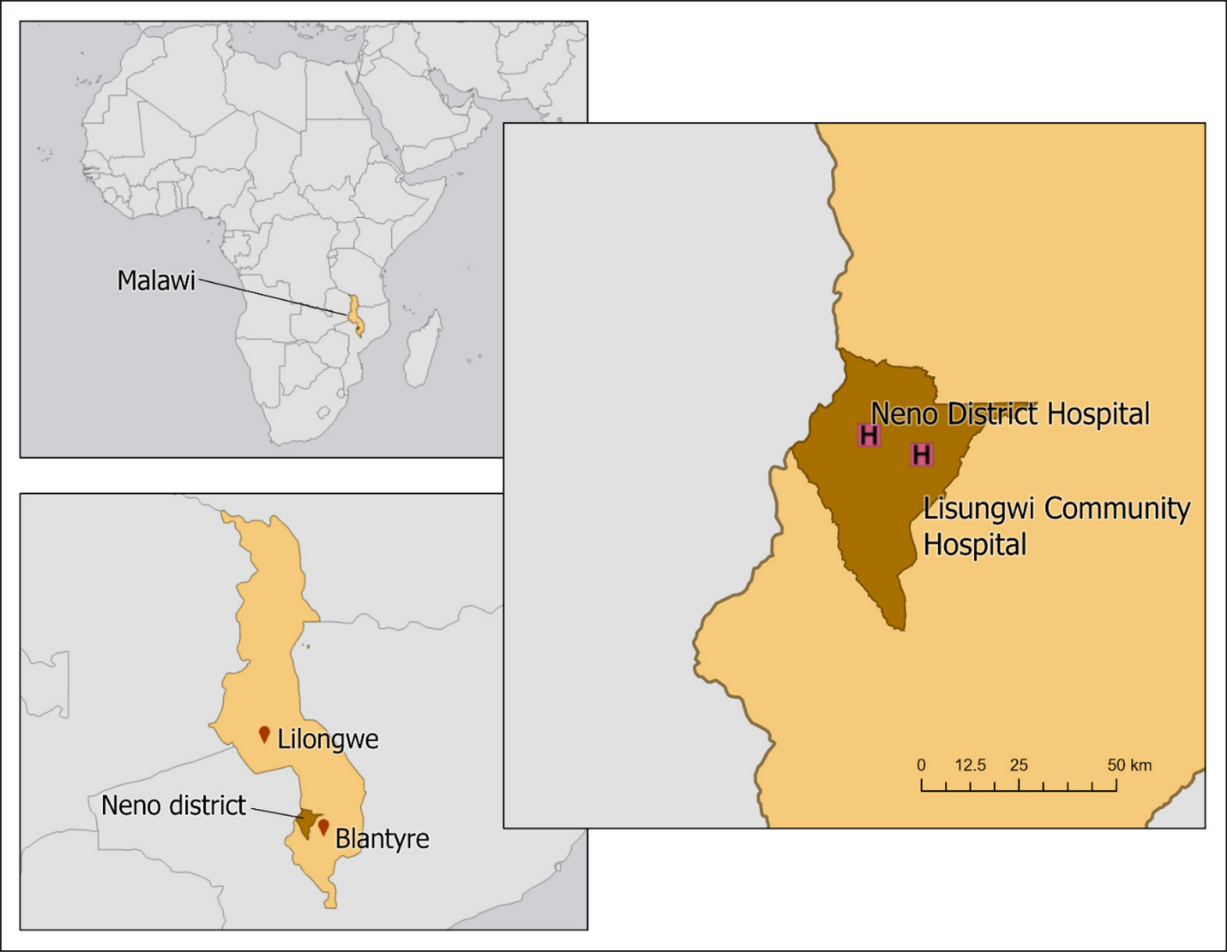
Map of Malawi illustrating the location of Neno district in relation to the two major urban areas and Neno’s two major health facilities where PEN-Plus clinics are located.

In 2015, the Ministry of Health and Partners In Health, locally known as Abwenzi Pa Za Umoyo, established an Integrated Chronic Care Clinic (IC3) [9] which offers decentralized care for common NCDs in all health facilities in Neno district [10], far removed from any urban area, consistent with the World Health Organization Package of Essential NCD Interventions [11]. Patients followed in IC3 have non-complex diagnoses such as hypertension, type 2 diabetes on oral therapy, and controlled asthma. In 2018, specialized clinics were established at the district hospital and Lisungwi community hospital (Figure 1), using a strategy called ‘PEN-Plus’ to provide outpatient treatment of more complex and severe NCDs such as HF, insulin-dependent diabetes, and sickle cell disease [12]. PEN-Plus uses a model of care decentralization for complex NCDs, provided by non-physician providers, thus making care more accessible to the rural poor.

The non-physician providers at PEN-Plus clinics are equipped with the knowledge, ongoing mentorship, and simplified algorithms for managing complex NCDs. Non-physician providers are involved in longitudinal patient care including initial evaluation, diagnosis, and categorization of HF, initiating treatment and establishing a follow-up plan, and linking patients requiring specialized care to specialists at a tertiary level of care.

There is limited data on cardiovascular disease epidemiology and burden available in Malawi, and none available in rural Malawi. An important step in improving the care of chronic HF in this population is understanding the burden of disease and the feasibility and clinical outcomes of a decentralized strategy led by non-physician providers. Our study aims to describe the etiology and clinical outcomes in patients presenting with clinical HF who are longitudinally enrolled in PEN-Plus clinics in rural Malawi.

## Methods

### Study setting and population

This study was conducted at two PEN-Plus clinics based at Lisungwi Community Hospital and Neno District Hospital (Figure 1). Neno district is in southwestern Malawi with a population of approximately 140,000 and very high rates of poverty [4]. The majority of the population relies on subsistence farming and only five percent have access to electricity [4]. Abwenzi Pa Za Umoyo has partnered with the Ministry of Health since 2007 to improve healthcare and support socio-economic development in the district.

The PEN-Plus clinics are managed by a team of clinical officers (non-physician providers), nurses, pharmacy technicians, and clerks with routine supervision by a general physician and nurse mentor. Clinical officers in Malawi undergo four years of medical training. Additionally, the PEN-Plus clinical officers in Neno district underwent three months of bedside training in FOCUS for HF diagnosis and training on managing HF. These non-physician providers also treat a range of other NCDs in PEN-Plus clinics, including type 1 diabetes, sickle cell disease, and severe hypertension. The additional training and management of more complex NCDs separates the PEN-Plus non-physician providers from those working in IC3, who do not have the additional training in management of severe NCDs.

### Heart failure diagnosis and initial management

Heart failure patients are identified in the inpatient wards, outpatient departments, or in IC3 using signs and symptoms as well as chest x-ray findings consistent with volume overload. Basic lab tests such as creatinine and electrolytes are mostly available, but B-type natriuretic peptide levels are not. Inpatient assessment and discharge planning are done by the ward team in conjunction with PEN-Plus providers. Upon discharge, medications are given and a follow-up appointment at the PEN-Plus clinic is assigned.

### Heart failure categorization and long-term management

**Figure 2:**
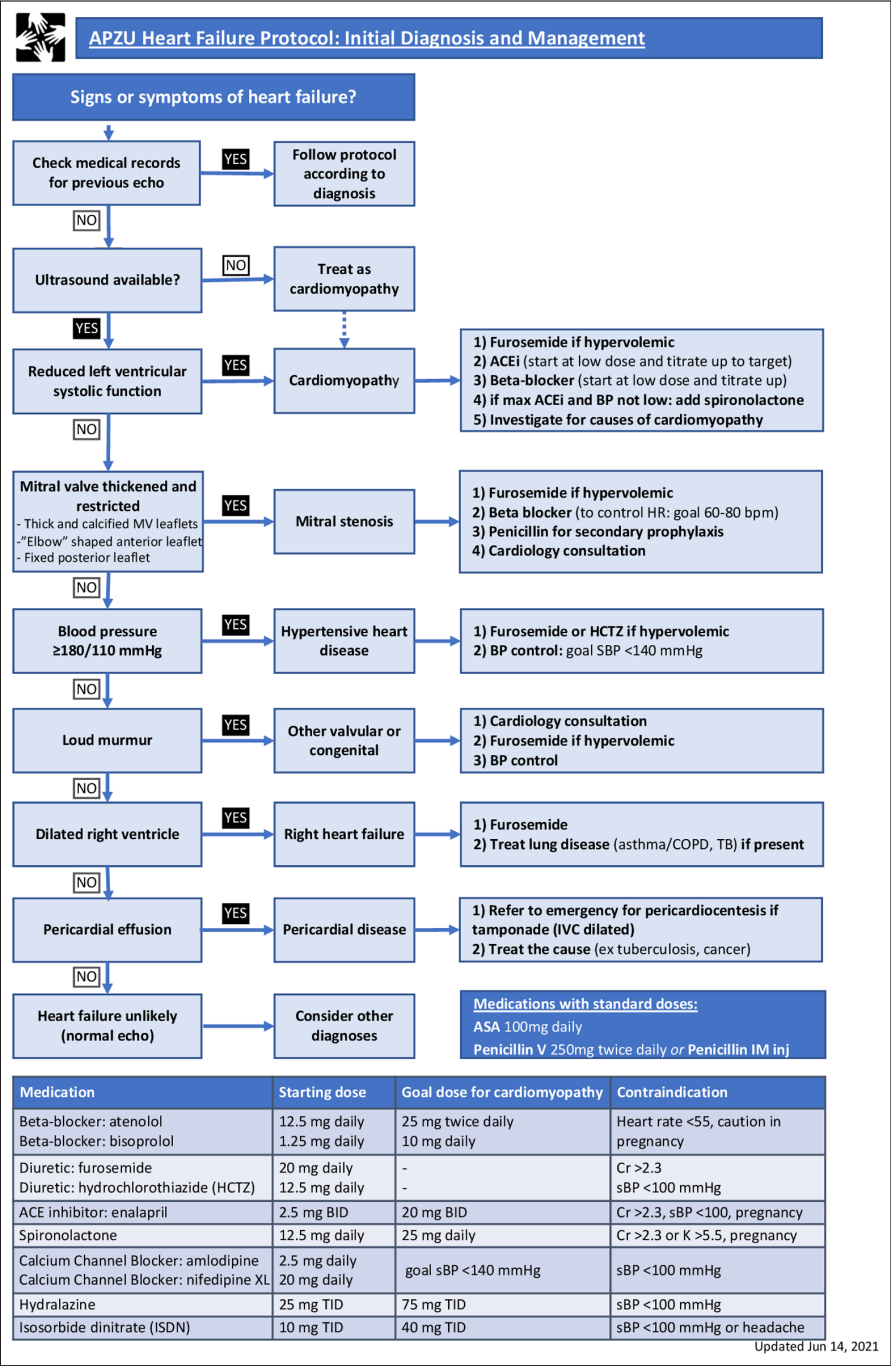
Heart failure protocol for initial diagnosis and management used by non-physician providers in PEN-Plus clinic in Neno, Malawi. Providers use focused cardiac ultrasound and follow the flow on the left-hand side of the protocol to determine a diagnostic category and management. The right-sided boxes guide management based on the diagnosis made.

An initial evaluation at the PEN-Plus clinic includes a complete history and physical examination. A focused cardiac ultrasound is performed [13] using a Sonosite M-Turbo portable ultrasound machine (FUJIFILM Sonosite, Bothell, WA), with cardiac 5–1 MHz probes, and interpreted by non-physician providers at the time of image acquisition to assign the patient into one of six diagnostic categories employing a simplified, evidence-based protocol (Figure 2).

In the protocol (Figure 2), non-physician providers are trained to classify visually reduced left ventricular contractility as cardiomyopathy. Further diagnostic testing on etiology of reduced left ventricular systolic function is limited though providers are trained to assess for common causes of cardiomyopathy such as HIV infection, alcohol consumption, and peripartum status. Rheumatic heart disease is identified using the fulfillment of two of three mitral valve criteria stated in the protocol. Other rheumatic valvular changes are felt to be above the level of non-physician providers in this setting and those that cause HF will likely cause an audible murmur, causing them to be classified in the ‘other valvular and congenital’ category. A dilated right ventricle is defined as a right ventricle larger than the left ventricle in the apical four-chamber view or a right ventricular outflow tract twice the diameter of the aortic root on the parasternal long-axis view. Hypertensive heart disease is defined by a blood pressure reading meeting criteria on enrollment, or an established history of hypertension. To simplify practice, non-physician providers were not trained to use color flow or spectral Doppler and did not diagnose specific valvular lesions other than rheumatic mitral stenosis. Measurements are not taught due to potential for measurement error and on design of the algorithm, it was felt that mild findings requiring measurement to confirm abnormality were unlikely to be causing significant clinical HF. Patients suspected to have rheumatic, valvular, or congenital heart disease were referred to a local pediatric cardiologist at the closest tertiary hospital, located 110 km away, for a full echocardiogram, guidance on management, and surgical evaluation if appropriate. Patients who could not afford to travel were supplied with transportation to facilitate the referral.

Each diagnostic category has a corresponding initial management plan to guide treatment with evidence-based medications and appropriate referral to specialists as outlined in the protocol (Figure 2). Diuretics are adjusted to relieve symptoms of congestion. Protocol recommendations for cardiomyopathy management are based on American cardiovascular guidelines [14]. Thus, patients can be started on evidence-based medications in the absence of specialists such as cardiologists. This protocol has been previously implemented and studied in rural Rwanda with demonstrated success. [15,16,17].

### Cardiologist verification of heart failure diagnosis

For study purposes, available cardiac ultrasound images were reviewed by board-certified cardiologists with specialization in echocardiography (SLK and GFK). Cardiologist interpretation was harmonized with non-physician providers’ interpretation. Following the flow of the diagnostic algorithm (Figure 2), cases of cardiomyopathy and mitral stenosis were confirmed to be present by cardiologist interpretation. When neither of those were present, blood pressure meeting criteria or an established history of hypertension according to non-physicians led to categorization as hypertensive heart disease. Cases where there were no apparent structural abnormalities on cardiac ultrasound and that were characterized by non-physician providers as valvular heart disease remained as such, given that the presence of a murmur was not recorded but likely auscultated during initial clinic evaluation. For cases with discrepant cardiologist and non-physician providers’ interpretations, most were labelled as ‘unable to classify’ given that cardiologists did not find structural heart abnormalities on the ultrasound images but ancillary clinical information may have been used to arrive at the diagnosis.

### Clinical follow-up

At all clinical visits, the NYHA class was documented, volume status was determined by physical examination, and medications were adjusted to target doses. Counselling on the diagnosis, medication adherence, lifestyle modification, and when to return to the hospital was provided. Follow-up visits are scheduled every one to three months, depending on the clinical severity.

To maximize patient retention, the PEN-Plus team performed home visits across Neno, for patients who missed scheduled appointments and those unable to attend PEN-Plus clinics due to long distances and cost of transportation. Home visits were performed for clinical evaluation and medication renewals. Older patients, patients with disability, and those with severe disease are prioritized for home visits.

The district is covered by a network of community health workers (CHWs) that visit a group of pre-assigned households [18]. CHWs report critical clinical outcomes directly to the IC3 and PEN-Plus clinics. Patients who do not present for follow-up visits are visited by their CHW to identify barriers to follow-up and re-enroll them into care. Mortality data is also reported through the CHW system.

### Design and data collection

Data from clinical charts in both PEN-Plus clinics between November 2018 and March 2021 was gathered retrospectively, including co-morbidities, clinical status at enrollment, FOCUS results, number of follow-up visits, and most recent clinical status at the time of chart review. Data was extracted by the primary author (BM) and electronic records were reviewed against paper charts to ensure accuracy. We performed a secondary review of the paper charts for instances of missing or discrepant data to ensure accuracy.

### Outcomes of Interest

Our primary outcome was the number of patients alive and actively followed at study closure. A patient was defined as lost to follow up when they were more than eight weeks late for their scheduled clinic appointment and attempts made to trace the patient were unsuccessful. Secondary outcomes were description of HF categories and change in patient clinical status from time of enrollment to most recent clinic visit: (1) change in signs and symptoms, and (2) change in NYHA class.

### Statistical analysis

We summarized categorical data as proportions and used Pearson Chi-square test or Fisher’s exact test to assess the association between two variables. We used the one-sample t-test to assess change in NYHA class between enrollment and follow-up among participants with available data. Loss to follow-up and mortality of patients stratified by NYHA class is described. We fitted the generalized linear model with binomial distribution to estimate the relative risk of death among NYHA class and further adjusted for sex and age. A p-value of <0.05 was considered significant. All data were analyzed using Stata version 15.1.

### Ethical Considerations

Ethical approval was obtained from the Malawi College of Medicine Research and Ethics committee (COMREC); Brigham and Women’s Hospital, Harvard Medical School; and the Neno district health research committee. All data collected in the study was part of routine clinical care in PEN-Plus clinics. Patient consent was waived due to the retrospective nature of the chart review.

## Results

### Demographics and clinical characteristics

There were 178 patients with HF enrolled in the PEN-Plus clinics, 103 (58%) women, and 18 (10%) children under age 18 years during the study period (Table 1). The median age at enrollment was 67 years (IQR 44 – 75), median body mass index was 20.4 kg/m^2^ and mean duration of follow-up in the clinic was 11.5 months (IQR 5.1–16.5). Hypertension was the most common comorbidity (44%), with prevalence slightly higher in women (57%) than men (42%). At enrollment, 33% had a systolic blood pressure of >140 mmHg, which decreased to 20% at follow up. Fourteen (8%) were HIV positive and on antiretroviral treatment.

**Table 1:** Patient Sociodemographic and clinical profiles.


CHARACTERISTIC	TOTAL POPULATION	MALE	FEMALE

**N (%)**	178	75 (42)	103(58)

**Median age at enrollment to the clinic – Years (n = 176)** ^*^	67 (IQR 44 –75)	71	60

**Mean duration of enrollment (months)**	11.5 (IQR 5.1–16.5)	11.0	11.5

**Median number of visits throughout the enrollment duration**	5 (IQR 3–9)	4	6

**Median BMI (kg/m** ^2^ **) (n = 153)**	20.4 (IQR 18.2–23.8)	19.8	20.2

Smoking status* (n = 174)	35 (20)	27 (77.1)	8 (22.9)

Alcohol* consumption (n = 174)	48 (28)	32 (66.7)	16 (33.3)

**HIV (n = 131)**	14 (11)	1 (7.1)	13 (92.9)

**Hypertension (n = 178)**	78 (44)	33 (42.3)	45 (57.7)

**Diabetes (n = 178)**	5 (3)	3 (60)	2 (40)


BMI: Body Mass Index; HIV: Human Immunodeficiency Virus. *Smoking status defined as actively smoking or stopped within past 12 months. Alcohol consumption defined as actively using alcohol at the time of enrollment.

### Signs and symptoms of heart failure

Upon enrollment in PEN-Plus clinics, more than 50% (n = 122) of the patients were symptomatic (NHYA II, III, or IV) and 64% were classified as NYHA II. Among the patients alive and in care at time of follow-up who had both baseline and follow-up data (n = 124), 2% were classified as NYHA III or IV. The proportion of patients with NYHA class II decreased from 61% to 48% (p < 0.0015), while the proportion of patients with NYHA class I increased from 24% to 50% (p < 0.001) (Table 2).

**Table 2:** Clinical profile at enrollment and follow-up N (%).


CHARACTERISTIC	ENROLLMENT	FOLLOW UP	P-VALUE*

**Orthopnea**	n = 127	n = 138	

**Yes**	93 (73)	12 (8)	0.010

**Cough**	n = 172	n = 136	

**Yes**	79 (46)	7 (5)	0.238

**Fatigue**	n = 172	n = 145	

**Yes**	83 (48)	9 (6)	0.001

**Chest Pain**	n = 172	n = 129	

**Yes**	66 (38)	3 (2)	0.072

**Peripheral Edema**	n = 176	n = 135	

**Yes**	66 (38)	12 (9)	0.001

**Bibasilar crackles**	n = 175	n = 133	

**Yes**	15(9)	5(4)	0.029

**JVP elevated**	n = 175	n = 125	

**Yes**	18 (10)	5 (4)	

**Volume status**	n = 157	n = 111	0.047

**Hypovolemic**	1 (1)	1 (1)	

**Euvolemic**	99 (63)	101 (91)	

**Hypervolemic**	57 (36)	9 (8)	

**NYHA class**	n = 159	n = 130	0.001

**NYHA I**	37 (23)	65 (50)	

**NYHA II**	101 (64)	61 (47)	

**NYHA III**	18 (11)	3 (2)	

**NYHA IV**	3 (2)	1 (1)	


JVP: jugular venous pressure; NYHA: New York Heart Association. *We computed and compared proportions for each symptom at enrollment and follow-up using chi-square and Fisher’s test.

### Heart failure categories

Cardiac ultrasound images for 154 patients were available for cardiologist review. The most common HF diagnostic categories were hypertensive heart disease in 56 (36.4%), cardiomyopathy in 40 (26.0%), and rheumatic mitral stenosis in 11 (7.1%) patients. There were 25 (16.2%) patients with other diagnoses or could not be classified (Table 3). There were 9 (5.8%) patients with a normal FOCUS scan. Isolated right HF could not be correlated with presence of lung disease based on chart review and limited available diagnostics.

**Table 3:** Heart failure diagnostic categories (n = 154).


DIAGNOSTIC CATEGORY	N (%)	MEAN AGE (SD)	PEDIATRIC PATIENTS <18, (N-15)%

**Hypertensive Heart Disease**	56 (36.4)	70.4 (12.0)	0

**Cardiomyopathy**	40 (26.0)	53.0 (20.2)	2

**Rheumatic Mitral stenosis**	11 (7.1)	28.8 (16.5)	4

**Other Valvular or Congenital Heart Disease**	8 (5.2)	15.3 (25.2)	6

**Right HF**	5 (3.3)	65.8 (20.2)	0

**Normal-focused cardiac ultrasound**	9 (5.8)	57.9 (14.8)	0

**Other/could not be classified**	25 (16.2)	50.9 (23.5)	3


### Clinical follow-up

By study closure, 50% of patients had attended five or more follow-up visits (IQR 3–9), 139 (78%) were alive and in care, 21 (12%) were lost to follow-up and 18 (10%) were known to have died. The crude relative risk of death was 3.94 for patients with enrollment NYHA III and IV vs. NYHA I & II (p < 0.005).

**Figure 3:**
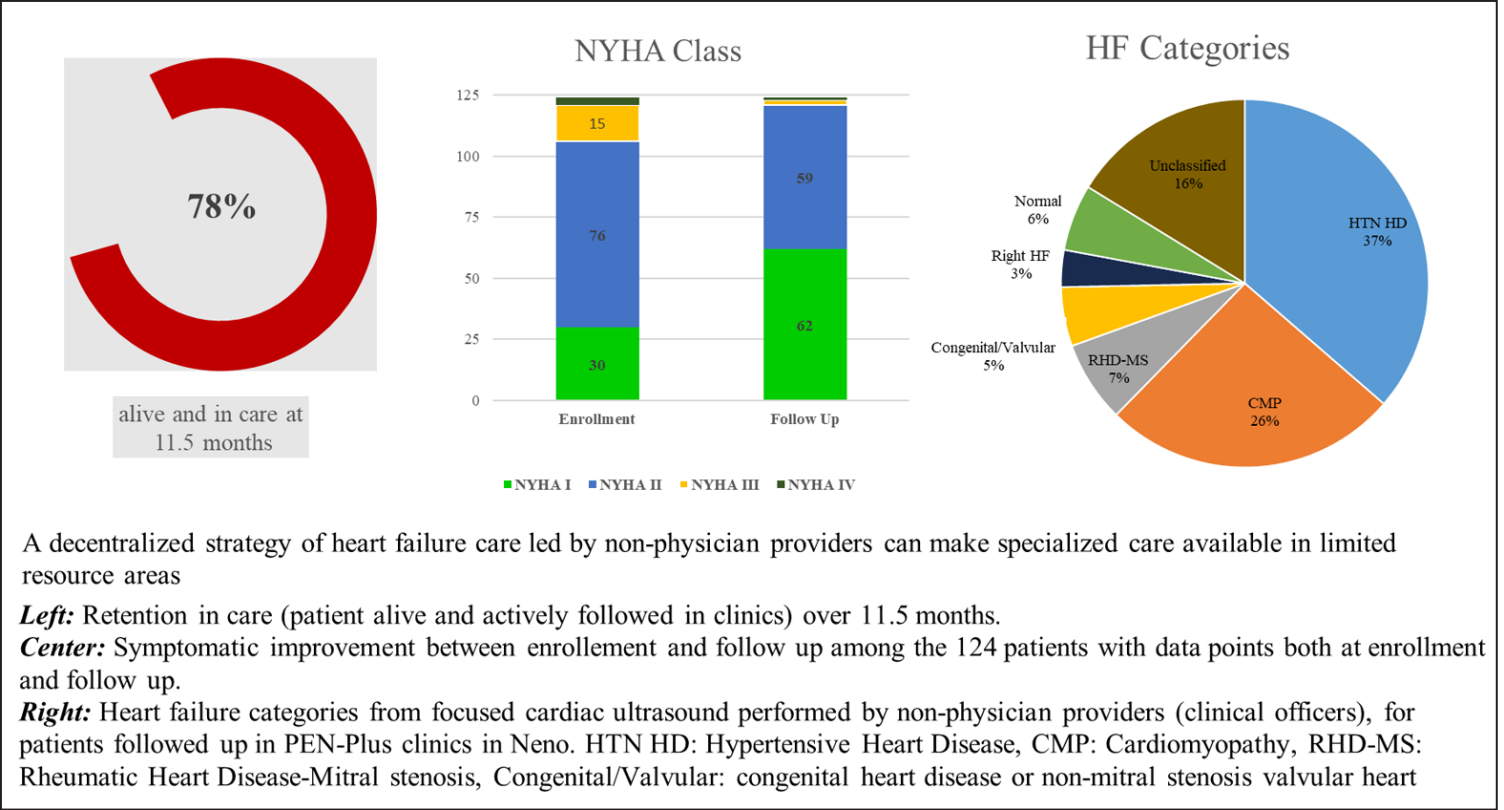
**Central Illustration:** Retention in care, New York Heart Association (NYHA) class improvement, and HF categories among patients managed by non-physician clinicians in Neno, rural Malawi.

Of the 124 patients that had documented NYHA class at both baseline and most recent follow-up, there was an improvement in symptoms (Figure 3). The proportion of patients with NYHA Class I symptoms increased from 24% to 50% (p < 0.001), while the proportion with NYHA Class II symptoms decreased from 61% to 48% (p < 0.0015), (Figure 3).

At follow up, symptoms of orthopnea, edema, fatigue, hypervolemia, and bibasilar crackles all decreased (p < 0.05) There was no statistically significant improvement in chest pain and cough. (Table 2).

## Discussion

In our retrospective study of patients enrolled with HF in PEN-Plus clinics in rural Malawi over a two year period, non-physician providers play an important role in longitudinal care and categorization of patients with HF. There was a high rate of retention in clinical follow-up in PEN-Plus clinic (78%) when compared with the general NCD population in the IC3 clinic (72%) [9]. In the studied cohort, we observe hypertensive heart disease (36.4%) and cardiomyopathy (26%) to be the most common causes of HF. We also describe significant improvement in HF symptoms, demonstrating further that non-physician-led clinics in this setting are feasible and effective in reducing symptoms and implementing HF management employing FOCUS as a diagnostic tool (Figure 3). Findings from our study support decentralization of cardiac care from tertiary facilities to rural health facilities using simplified clinical algorithms and targeted training of non-physician staff. This may be a clinic process that could be disseminated to other clinics in sub-Saharan Africa.

Our retention in care (78%), was higher when compared to implementation of a similar model of care in Rwanda which had a retention rate of 42.8%. [19]. A major factor in our high retention rates is likely the shorter average length of follow-up (11.5 months) for the Malawian cohort as compared with patients followed in Rwanda (27.6 months). Additionally, home visits and the provision of social support in the form of transport reimbursements made it possible for patients to keep their appointments given the high poverty rate in rural Malawi [20].

Regarding the causes of HF, our data differs from that of urban Malawi where 34% of HF was caused by rheumatic heart disease, 24% from hypertensive heart disease, and 19% from cardiomyopathy [3]. In rural Rwanda, where a similar decentralized PEN-Plus approach was deployed, 54% of HF among adults was caused by cardiomyopathy and 25% was rheumatic heart disease. In a similar Rwandan population, 48% of HF in children was caused by rheumatic heart disease and 39% was from congenital heart disease [21]. The predominance of rheumatic heart disease in the Malawi urban setting may be from differing causes such as referral bias of younger patient cohorts to a cardiologist-run clinic, differences in housing, and access to primary healthcare in Neno district. In Rwanda, the studied cohort was composed of a high percentage of pediatric patients, possibly leading to more prevalent rheumatic heart disease [21].

Our loss to follow-up (12%) was lower than the loss to follow-up reported in IC3 clinic (25.5% over a 36-month follow-up period) even though both clinics are based in the same district [9]. This could be related to wider coverage of IC3 (in all health facilities in Neno), making it accessible to people outside Neno catchment area which are not covered by CHWs. Additionally, we believe that symptomatic patients followed in PEN-Plus are likely to stay engaged in care, and home visits contributed to increasing retention in PEN-Plus clinic [22].

Our rate of known deaths at 10% was higher than that of IC3, which was 1.4% [9], but lower than the one year HF mortality in other sub-Saharan Africa countries which has been reported at 22.5 – 50%. [23,24]. The higher death rate in PEN-Plus clinics compared to IC3 is likely due to the enrollment of complex NCDs, as opposed to less severe NCDs in IC3. We also believe that the death rate in our cohort may be affected by late presentation of cardiac disease in the rural setting, lack of access to cardiac surgery, and barriers such as distance and travel by foot which may have affected clinic attendance.

While our death rate is low when compared to other sub-Sahara African (SSA) countries, it is worth noting the difference in setting. Previous studies on an in-patient population [25,26,27,28,29] revealed a higher death rate compared to our outpatient model where enrolled patients are those who survive admission and are healthy enough to come to the clinic. Our model incorporates community-based care systems that improve enrollment in care, early detection, and referral for care in cases of clinical deterioration. Additionally, the provision of social support and home visitation by a dedicated team may be additional reasons for good outcomes. When compared with clinics implementing similar interventions, our loss to follow up was lower compared to the 29% rate in Rwanda, while deaths were similar at 9% [21]. Our overall low loss to follow-up and death rate underscores the feasibility and success of the PEN-Plus strategy.

Our enrollment data showed small numbers of patients with peripheral edema and jugular venous distension, which may be due to patients being treated and stabilized in the hospital before enrollment into PEN-Plus clinics. This is suspected to underestimate the clinical severity of HF at the initial clinic enrollment visit.

Out of 154 available FOCUS images, nine (5.8%) images were classified as normal despite presenting with clinical signs and symptoms of HF. This may be due to the nature of FOCUS, which does not incorporate more nuanced causes of HF such as diastolic dysfunction and constrictive pericarditis. In cases of valvular heart disease, when the murmur is soft or diastolic, these may be miscategorized as normal. In addition, due to limited diagnostic capacity such as absence of brain natriuretic peptide, patients may be misdiagnosed as having HF when their symptoms and physical exam findings are attributable to another cause.

Due to the retrospective nature of our study and the limited-resource setting, there were several limitations of note. Cardiologists had access to and reviewed 154 images of the 178 enrolled HF patients. Images for the remaining 24 patients were unavailable and the diagnosis made by non-physician providers may have been inaccurate. Further study is required to assess the accuracy of clinicians in interpreting the images compared to cardiologist interpretation and evaluate quality of the care delivered. Our study described the implementation of a PEN-Plus clinic in Neno. No pre-implementation data or comparison group was available, thus the absolute effect of PEN-Plus clinics compared to usual care in rural Malawi could not be determined. Incomplete patient charting due to inconsistent staffing and other resource challenges affected data integrity and was present in all clinics. Heart failure categories were not confirmed by a cardiologist in real-time, thus broad categories such as ‘valvular and congenital heart disease’ were used with the recommendation to refer for cardiologist assessment. Our study sites were supported by a non-governmental organization (Abwenzi Pa Za Umoyo) with added social and financial support for patients. Dissemination of a PEN-Plus strategy including HF care to other regions of Malawi or other LMICs may face resource challenges not present at our site.

## Conclusion

Using a decentralized strategy of HF care led by non-physician providers, the PEN-Plus approach can make specialized cardiac care available to the rural poor in Malawi. A favorable retention in chronic care was seen in the setting of integration with community-based care. The common causes of HF are similar to rural regions of other LMICs including hypertensive heart disease, cardiomyopathy, and rheumatic heart disease. Longer term patient outcome and provider training data will help to refine the PEN-Plus approach and ensure that all rural patients can be reached. Contextually-adapted implementation of similarly integrated HF care strategies through training a decentralized workforce could further improve equity and accessibility to cardiac care in rural areas in sub-Saharan Africa.

Endnote: Resources for PEN-Plus clinic implementation can be found at http://www.ncdipoverty.org.

## Key Messages

**What is already known on this topic:** Heart failure has been described in the Malawian urban setting, but in the majority rural population, HF data is scarce.

**What this study adds:** In our retrospective study, of patients enrolled in PEN-Plus clinics in rural Malawi over a two year period, there was a high rate of retention in clinical follow-up and significant improvement in HF symptoms demonstrating that non-physician-led clinics in this setting are feasible and effective in reducing symptoms and implementing HF management employing FOCUS as a diagnostic tool.

**How this study might affect research, practice, or policy:** Findings from our study support the decentralization of cardiac care from tertiary facilities to rural health facilities using simplified clinical algorithms and targeted training of non-physician providers. This approach, part of the PEN-Plus strategy, may be generalized to other clinics in sub-Saharan Africa.

## Abbreviations

- CHW – Community Health Workers
- NCD – Non-communicable disease
